# Cutaneous Calcified Mass of Foot in Pseudohypoparathyoidism: Case Report

**DOI:** 10.3390/medicina60040595

**Published:** 2024-04-04

**Authors:** Sang Heon Lee, Sung Hwan Kim, Seung Jin Choi, Young Koo Lee

**Affiliations:** Department of Orthopaedic Surgery, Soonchunhyang University Hospital Bucheon, 170, Jomaru-ro, Wonmi-gu, Gyeonggi-do, Bucheon-si 14584, Republic of Korea; worldking70@naver.com (S.H.L.); shk9528@naver.com (S.H.K.); 142178@schmc.ac.kr (S.J.C.)

**Keywords:** calcification, parathyroid hormone, pseudohypoparathyroidism

## Abstract

Soft tissue calcifications frequently appear on imaging studies, representing a prevalent but non-specific discovery, varying from a local reaction without clear cause to suggesting an underlying systemic condition. Because calcifications like these can arise from various causes, an accurate differential diagnosis is crucial. Differential diagnosis entails a methodical assessment of the patient, encompassing clinical presentation, medical history, radiological and pathological findings, and other pertinent factors. Through scrutiny of the patient’s medical and trauma history, we can refine potential causes of calcification to vascular, metabolic, autoimmune, neoplastic, or traumatic origins. Furthermore, routine laboratory assessments, including serum levels of calcium, phosphorus, ionized calcium, vitamin D metabolites, and parathyroid hormone (PTH), aid in identifying metabolic etiologies. We describe a rare occurrence of osteoma cutis in a 15-year-old female patient with a history of pseudohypoparathyroidism (PHP) and Albright’s hereditary osteodystrophy (AHO). The patient presented with a painful mass on the lateral side of her left foot. The diagnosis was based on medical history, laboratory tests, and imaging, leading to an excisional biopsy and complete pain relief post-surgery. Understanding such rare occurrences and related conditions is crucial for accurate diagnosis and management.

## 1. Introduction

The parathyroid gland is composed of four glands, which secrete the protein hormone parathyroid hormone. Parathyroid hormone functions to inhibit renal tubular reabsorption of phosphate, and increase renal excretion of phosphate, decrease plasma phosphate concentration [1]. It also demineralizes bone and increases renal tubular reabsorption of calcium to increase plasma calcium concentration [2]. PTH (parathyroid hormone) induces an increase in the number of bone-forming cells by promoting the growth of osteoblasts and reducing osteoblast cell death or apoptosis [3]. PTH also stimulates osteoclastogenesis. Mice lacking osteoclasts do not exhibit a response to PTH, indicating that osteoclast activity is necessary for PTH to exert its full anabolic effects [4]. Moreover, PTH regulates specific skeletal growth factors, such as IGF-1, and growth factor antagonists, like sclerostin, to further enhance the process of bone formation [5]. The osteoanabolic effect of PTH is utilized in its administration to humans for the treatment of severe osteoporosis, and this therapeutic window is commonly referred to as the “anabolic window” [6]. Daily injections of PTH have proven to be an effective FDA-approved treatment for osteoporosis, leading to significant improvements in both bone mineral density and bone volume [7].

Hypoparathyroidism is a condition characterized by the atrophy or absence of the parathyroid gland due to autoantibodies, resulting in low hormone levels in the bloodstream and hypocalcemia. This disorder is characterized by significantly reduced bone remodeling caused by the absence or significant reduction of PTH [8]. A chronic reduction in bone turnover in individuals with hypoparathyroidism usually results in increased bone mass compared to age- and sex-matched controls. 

Pseudohypoparathyroidism (PHP), which is historically the first hormone resistance syndrome, is a metabolic disorder first described by Albright in 1942 [9]. In this condition, parathyroid hormone secretion is normal, but there is abnormal responsiveness of target cells in bones and kidneys to the hormone. PHP comprises four types, with the most common form being PHP type Ia, which is often accompanied by Albright’s hereditary osteodystrophy (AHO). These are uncommon hereditary metabolic conditions; the prevalence is estimated to be around 0.79 per 100,000 [10]. The constellation of developmental and skeletal defects in AHO includes short stature, round faces, shortened fourth metacarpals and other bones of the hands and feet, obesity, dental hypoplasia, and osteoma cutis [1]. The initial symptom most commonly manifests as seizures around the age of 8 [11].

A 15-year-old woman, diagnosed and undergoing treatment for pseudohypoparathyroidism in her medical history, suffered from a painful calcified mass on her left foot. The authors aim to report a rare case of cutaneous ossification identified on histological examination after excising the mass.

## 2. Case Presentation

### 2.1. Preoperative Evaluation

**Figure 1:**
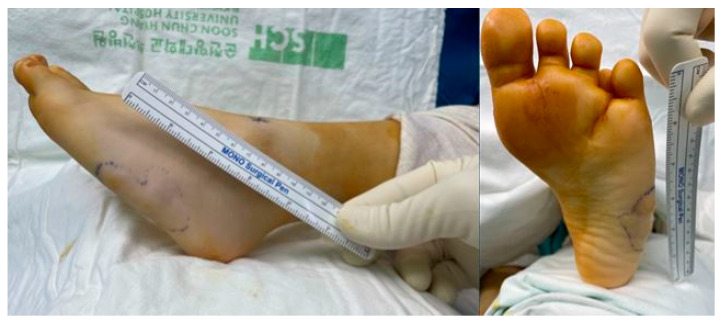
Preoperative findings. Fixed, non-mobile, 5 × 4 sized hard mass was noted on lateral side of left foot.

**Figure 2:**
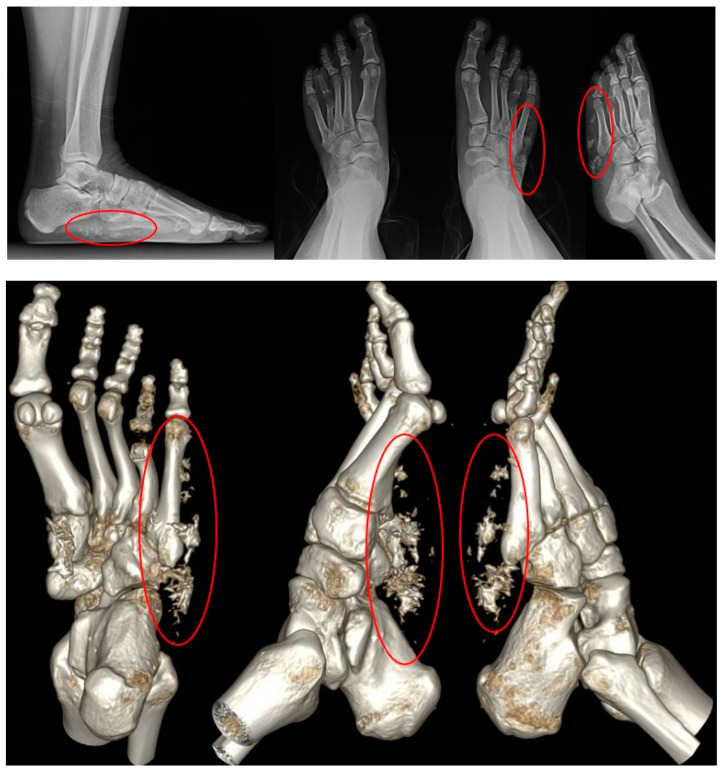
Preoperative X-ray (**Above**) and CT (**Below**). Soft tissue calcification was noted on the lateral side of left foot and brachydactyly of the fourth metatarsal bones was noted in both feet.

**Figure 3:**
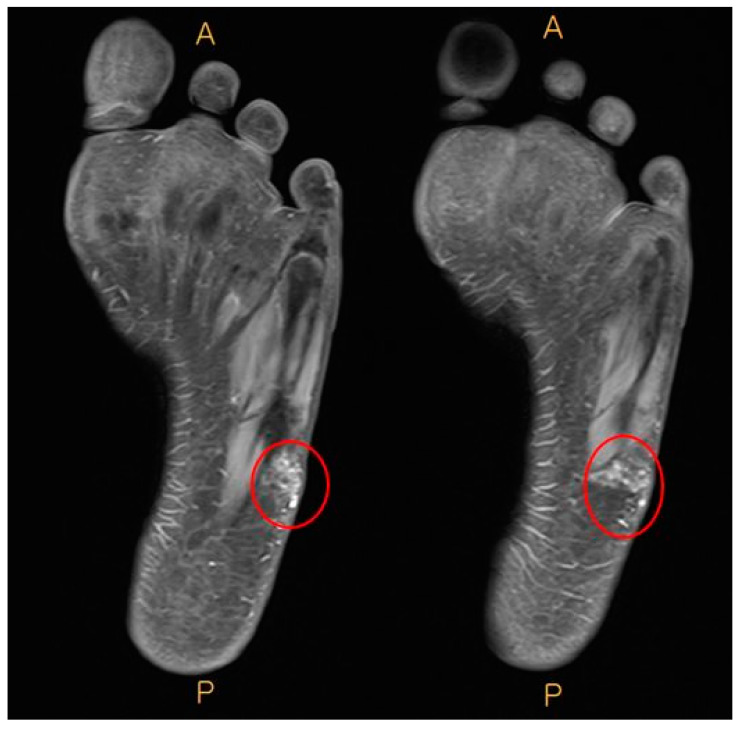
Preoperative contrast enhanced MRI. Enhanced high signal mass-like was observed on the lateral side of left foot. (A: Anterior, P: Posterior)

A 15-year-old woman presented to the clinic with a mass on the lateral side of her left foot, accompanied by pain. She reports no significant trauma to the area and mentions that the mass on her left foot began to be noticed approximately 10 years ago. From the onset of the palpable mass, the patient experienced accompanying pain, and over time, it gradually increased in size. Upon presentation, a hard mass with an irregular border, measuring 5 × 4 cm size, was palpated on the lateral side of the left foot. The surrounding tissues appeared fixed, and the mass was non-mobile, causing pain (Figure 1). The preoperative AOFAS (American Orthopaedic Foot & Ankle Society) and VAS (Visual Analogue Scale) scores were 40 and 7, respectively. On X-ray and CT examination, irregularly scattered calcifications were observed in the subcutaneous tissues on the lateral side of the left foot. Additionally, evidence of brachydactyly of the fourth metatarsal bones was noted in both feet (Figure 2). On contrast-enhanced MRI, a high-signal mass-like lesion enhanced with contrast was observed in the same area as in CT (Figure 3). She underwent an excisional biopsy for a firm mass with associated pain on the left wrist five months ago. At that time, X-ray findings indicated brachydactyly of the first and fourth metacarpal bones, and histopathological analysis of the mass confirmed the diagnosis of osteoma cutis.

**Figure 4:**
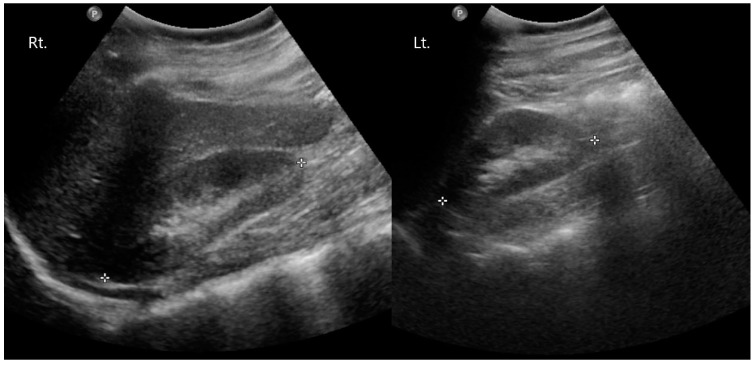
Preoperative kidney ultrasonography (USG). No abnormal findings were observed.

**Figure 5:**
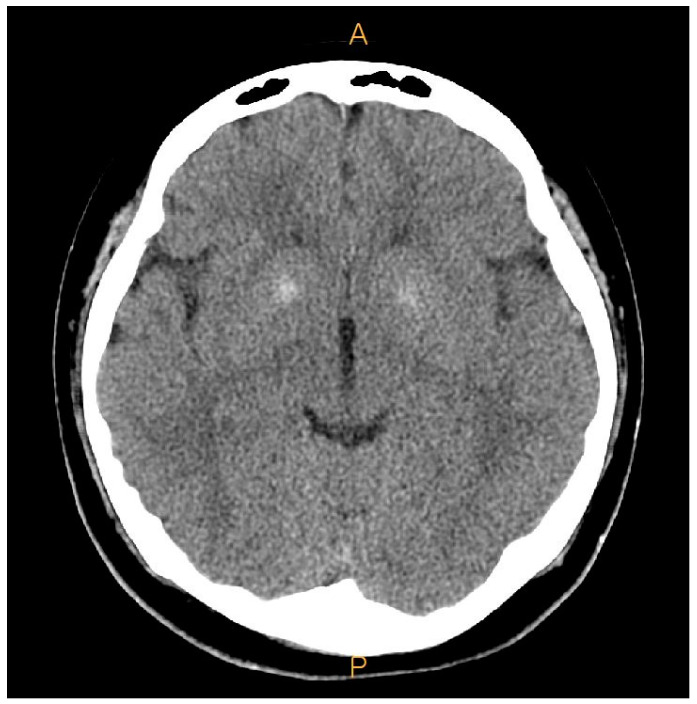
Preoperative brain computer tomography (CT). Calcifications were noted at both basal ganglia. (A: Anterior, P: Posterior)

The patient has a past medical history of being diagnosed with congenital hypothyroidism during newborn screening at seven days old and has received thyroid hormone replacement therapy. She was diagnosed with AHO and PHP through genetic sequencing at another hospital twelve years ago. To determine whether there were mutations in the GNAS gene of this patient, the nucleotide sequences of 13 exons and exon-intron boundaries of the GNAS gene were analyzed. The nucleotide sequence analysis revealed that the patient exhibited a heterozygous c.348_349ins(C) insertion mutation in exon 5 of the GNAS gene. This mutation has been reported in patients with PHP 1a. At the time of the initial diagnosis of PHP, blood tests revealed dyslipidemia, and there was hyperphosphatemia with a phosphorus concentration of 7.2 mg/dL (reference range: 2.5–4.5 mg/dL). The calcium concentration was within the normal range at 9.4 mg/dL (reference range: 8.3–10.0 mg/dL). PTH was elevated at 643.03 pg/mL (reference range: 8–76 pg/mL), thyroid stimulating hormone (TSH) was within the normal range at 0.49 uIU/mL (reference range: 0.25–4.0 uIU/mL), serum creatinine level showed a slight decrease to 0.4 mg/dL (reference range: 0.5–1.2 mg/dL) and 25-(OH) vitamin D3 was decreased at 21.80 ng/mL (reference range: 30–100 ng/mL). At that time, other typical signs, such as a round face and short stature, were not present. She has been undergoing outpatient follow-up and observation at the pediatric department of our hospital. During this time, she has been receiving medication therapy, including calcium supplementation (Calcium carbonate^®^, Nexpharm Korea, Daejeon, Republic of Korea, 500 mg/day), vitamin D supplementation (Bonky soft cap, YuYu Pharm, Seoul, Republic of Korea, 0.25 mcg/day), and thyroid hormone replacement therapy (Synthyroid, Bukwang Pharm, Seoul, Republic of Korea, 0.05 mg/day). A kidney ultrasound was performed, and no abnormal findings were observed (Figure 4). At the time of the first visit to the orthopedic department, the phosphorus concentration was 4.9 mg/dL, the calcium concentration was 8.1 mg/dL, PTH was elevated at 619.50 pg/mL, and TSH was within the normal range at 2.02 uIU/mL. Before surgery, calcium levels were found to be within the normal range at 9.1 mg/dL, while phosphorus levels were elevated at 5.8 mg/dL and serum creatinine was within the normal range at 0.9 mg/dL. A year ago, on a Brain CT, calcifications were observed in both basal ganglia without any accompanying seizures or neurological symptoms (Figure 5). She had a normal face appearance and short body stature. Her height was 149 cm and her weight was 54 kg, resulting in a BMI of 24.01. Her parents also underwent genetic testing, and no mutations were found. However, her mother had diabetes and hypothyroidism. 

### 2.2. Surgical Procedure

**Figure 6:**
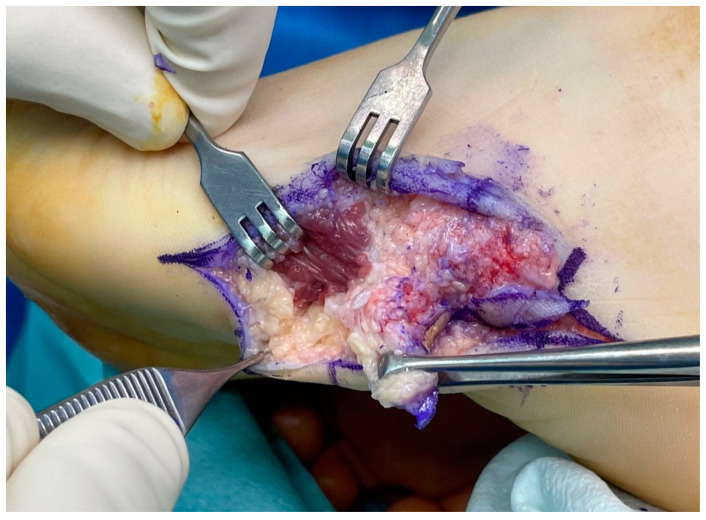
Intra-operative findings. Calcified tissue was noted to extend to the margin of the abductor digiti minimi muscle.

**Figure 7:**
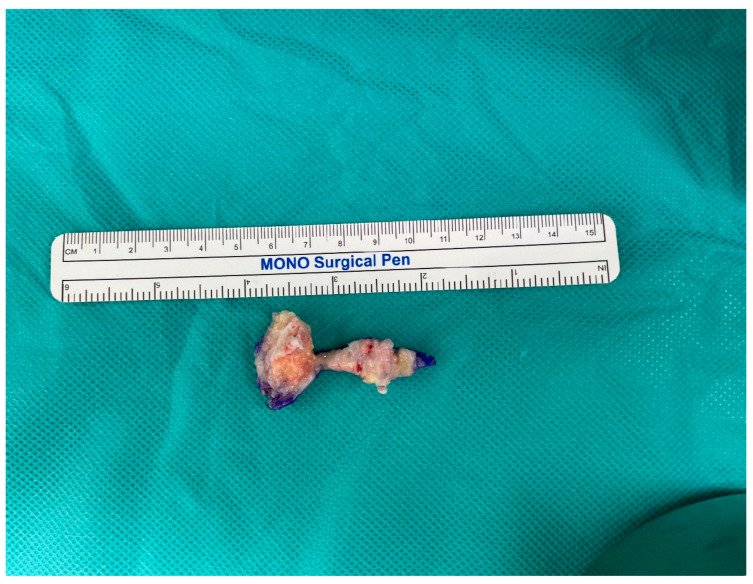
Photograph of specimen. Irregularly shaped 5 × 3 × 2 cm-sized calcified hard mass was excised.

The patient underwent an excisional biopsy of the mass on the left foot. Under general anesthesia, she was laid in the supine position and applied the pneumatic tourniquet at 300 mmHg. We applied betadine on the left lower extremity as part of skin preparation, and routine draping was performed. And the pneumatic tourniquet was inflated. A transverse 6 cm incision was made on the lateral side of her left foot around the mass. After dissecting soft tissue, we identified the mass that was firmly fixed to the surrounding tissues and non-mobile, with calcified tissue observed extending up to the border of the abductor digiti minimi muscle (Figure 6). The excised mass appeared as a hard, irregularly shaped calcified tissue measuring 5 × 3 × 2 cm on gross examination (Figure 7). 

### 2.3. Postoperative Care

Post-operative pathology confirmed cutaneous ossification. Following the surgery, the pain at the site of the mass disappeared completely. The AOFAS and VAS scores were 100 and 0, respectively, 1 year after surgery (Table 1). There were no impairments in walking or daily activities, indicating a successful recovery. The patient, who was initially under the care of the Department of Pediatrics at our institution, continues to undergo endocrinological evaluations at the same department following surgery.

## 3. Discussion

PTH is the primary regulator of serum calcium, primarily acting on the kidney and bone through its Gs-coupled receptor, PTHR1. It binds to parathyroid hormone receptors on the cell membrane, activating G-proteins (guanine nucleotide-binding proteins), which in turn activate adenylate cyclase to produce cyclic AMP (cAMP) as a secondary messenger, thus mediating the hormone’s functions [12,13]. In bones, it promotes the release of calcium and phosphate into the bloodstream, while in the kidneys, it stimulates calcium reabsorption and inhibits phosphate reabsorption, resulting in phosphaturia. In the renal proximal tubule, PTH stimulates the transcription of the 25-hydroxyvitamin D 1-α hydroxylase gene, resulting in the production of active 1,25-dihydroxyvitamin D3 (1,25-(OH)2D). This, in turn, enhances the absorption of calcium and phosphate from the bowel. Therefore, overall, PTH is a hormone that increases serum calcium levels and decreases phosphate levels in the blood. 

PHP is a hereditary disorder characterized by symptoms and signs of hypoparathyroidism, particularly abnormalities in the skeletal system. It is classified into four types based on the types of organs and molecular biological abnormalities exhibiting resistance. These types include abnormalities in G-proteins for Type Ia, receptor abnormalities for parathyroid hormone in Type Ib, abnormalities in adenylate cyclase for Type Ic, abnormalities in intracellular signal transduction following the formation of secondary messengers for Type II, and a variant of Type I known as pseudopseudohypoparathyroidism (PPHP) [10]. 

AHO, initially described alongside PHP in 1942, arises from a heterozygous mutation in the GNAS gene, which encodes the G-stimulatory subunit (Gαs) of the guanine nucleotide-binding protein. Gαs is responsible for cyclic AMP generation, a critical mediator for hormonal actions. Thus, mutations in GNAS that result in the loss of expression or function of the G-stimulatory subunit restrict cyclic AMP generation, leading to an imprinting disorder [14]. It represents a clinical entity characterized by heterogeneous clinical manifestations, including brachydactyly, a rounded face, short stature, central obesity, subcutaneous ossifications, and varying degrees of mental retardation. Blood tests reveal hypocalcemia and hyperphosphatemia, along with an increase in parathyroid hormone levels. Symptoms of hypocalcemia typically present with stiffness and seizures, particularly around the age of 8. In particular, brachydactyly, which is characterized by the shortening of the third, fourth, and fifth metacarpals and the distal phalanx of the first finger, is a typical and highly specific feature of the AHO phenotype, along with heterotopic ossifications [15]. Patients with PHP often experience chronic hypocalcemia, leading to calcification of the basal ganglia, cataracts, and other manifestations. Approximately 50% of patients with PHP exhibit basal ganglia calcification, and various factors are presumed to act on the pathogenesis of brain calcification before its onset. Plasma phosphate overload is considered the primary mechanism [16,17]. Calcified nodules are frequently observed during physical examinations in patients with AHO. These nodules represent genuine heterotopic intramembranous ossifications, typically confined to the subcutaneous tissues. The number and extent of these nodules vary widely among individuals with AHO. 

Osteoma cutis, or cutaneous ossification, is defined by the development of morphologically normal bone within the dermis or subcutaneous tissue. It accounts for 14% of all cutaneous ossifications. And there is a female predominance in osteoma cutis, with a peak incidence in the second and third decades [18]. When this condition arises spontaneously, without a pre-existing condition, it is classified as primary osteoma cutis [19]. Secondary ossification occurs in association with underlying inflammatory, neoplastic, or traumatic conditions, and it is more common than primary osteoma cutis [20]. Secondary osteomas account for 85% of cutaneous ossifications and primary osteoma cutis accounts for about 15% of cutaneous ossifications [21]. As we can see in our case, primary osteoma cutis is also seen in Albright’s hereditary osteodystrophy (AHO) with pseudohypoparathyroidism (PHP). It primarily manifests in periarticular regions such as the scalp, hands, and feet, but it can occur anywhere on the body [22]. The mechanism of bone formation in the skin is not fully understood. One theory suggests that it occurs through the metaplasia of fibroblasts into osteoblasts due to alterations in the genes regulating bone formation. Factors such as bone morphogenetic proteins and growth factors, which are expressed in the skin, may induce the differentiation of fibroblasts into osteoblast-like cells. These cells can then initiate osteogenic changes in the skin, leading to bone formation [23]. The prognosis of osteoma cutis is generally favorable, with most cases presenting as benign skin lesions that can be managed effectively with excision. However, patients should undergo monitoring for associated conditions that may lead to hormone resistance and more significant cosmetic defects. Progressive osseous heteroplasia (POH), on the other hand, represents a disabling disease. POH is part of a spectrum of related genetic disorders, which includes AHO, PHP, and primary osteoma cutis. These disorders share common features of superficial ossification and are associated with inactivating mutations of the GNAS gene [24]. While it may initially present with cutaneous ossification in childhood, it progresses to involve deep skeletal muscle [25].

On imaging examinations, the presence of soft-tissue calcifications is a common yet nonspecific finding, which can range from a nonspecific local reaction to indicating a systemic condition [26]. Classically, these calcifications are categorized into four types based on the mechanism of formation as well as clinical and biochemical correlation: dystrophic, metastatic (metabolic), idiopathic, and iatrogenic [27]. Dystrophic and iatrogenic calcifications arise in damaged or degenerated tissue and constitute approximately 95–98% of all soft-tissue calcifications. Dystrophic calcifications are associated with tissue damage or degeneration, while iatrogenic calcifications are correlated with surgical manipulation or medication infusion. Metastatic (metabolic) calcifications result from systemic metabolic disorders, and the most common cause is end-stage renal disease. As calcification typically occurs when there is an association of hypercalcemia with hyperphosphatemia, it can indeed happen due to secondary hyperparathyroidism in patients with renal failure. Other causes include disorders of calcium and phosphate metabolism, including hypoparathyroidism and PHP. Gout, which arises from hyperuricemia, is another potential cause of such calcifications [28]. Idiopathic calcifications can occur in tumoral calcinosis, a rare familial disease characterized by abnormal regulation of phosphate metabolism [29]. Because of these various potential causes that can lead to calcification, differential diagnosis is crucial above all. Differential diagnosis is achieved through systematic evaluation of the patient, including clinical presentation, history, radiological findings, pathological findings, and other relevant factors. By assessing the patient’s medical history and trauma history, among other factors, we can narrow down the potential causes of calcification to vascular, metabolic, autoimmune, neoplastic, or traumatic origins. Not only by investigating the patient’s history, but also routine laboratory measurements of serum calcium, phosphorus, and ionized serum calcium, vitamin D metabolites, and PTH should be performed to identify metabolic causes [30]. 

Calcific deposits, although of metabolic origin like PHP and AHO, could potentially cause changes in the patient’s gait pattern due to the mass itself, leading to alterations in walking. These gait disturbances could potentially contribute to the onset and exacerbation of foot pain. While there is not extensive research or cases specifically addressing the alteration in plantar pressure patterns due to calcified masses on the foot, it’s conceivable that patients with foot pathologies like gout or Sever’s disease may exhibit issues with plantar pressure distribution, resulting in abnormal gait patterns [31,32]. In such cases, employing methods such as wearing footwear with good characteristics can help alleviate pain by reducing abnormal plantar pressure [33]. Similar strategies could potentially be effective in patients with foot-calcified masses, like the one in this case, by mitigating the effects of altered plantar pressure distribution through appropriate footwear.

In this case, considering the patient’s medical history and the results of TSH and phosphate levels in the blood tests, narrowing down the cause of calcification to a metabolic origin appears to be reasonable. With clues such as brachydactyly of both 4th metatarsal bones and a history of AHO, the diagnosis of ectopic ossification due to PHP could be made. When a patient presents with masses accompanied by calcification in various parts of the body, it’s important to understand that there are numerous conditions that can cause calcification. Through a systematic evaluation of the patient, a differential diagnosis can be made, and further, anticipating the multitude of complications that may arise from the underlying condition is crucial. This allows for the formulation of treatment plans that take into account potential complications in advance.

We have searched three other cases for comparison with this case. In two cases, the patients were diagnosed with PHP type 1a, while the remaining case was diagnosed with PPHP. We have compiled the similarities and differences of each case into a table for comparison (Table 2) [2,34,35].

## 4. Conclusions

We describe a rare case of osteoma cutis occurring in a 15-year-old female patient with a history of PHP and AHO. Her medical history, laboratory test results, and X-rays allowed us to diagnose osteoma cutis due to PHP. After the excisional biopsy, the mass did not recur, and there was complete relief of pain, with no discomfort reported. 

## Figures and Tables

**Table 1:** Clinical outcomes comparing before and after surgery.

Variables	Preop	POD 6m	POD 1yr
AOFAS	40	90	100
VAS	7	2	0
FAOS	136	62	42

AOFAS: American Orthopedic Foot and Ankle Society, VAS: Visual Analog Scale, FAOS: Foot and Ankle Outcome Score, Preop: Preoperative score, POD 6m: Postoperative 6month score, POD 1yr: Postoperative 1year score.

**Table 2:** Comparison between other three cases.

Study	This Case	X.L. Wang [32]	G. Sethuraman et al. [33]	Ki-Heon Jeong et al. [2]
Sex/Age (years)	F/15	M/16	M/7	F/11
Location	Lat. side of Lt. foot	Lat. side of Lt. 2nd & 3rd finger	Abdomen & extremities	Rt. palm, Lt. 5th finger, Lt. sole
Symptom	Lt. foot pain	Lt. finger pain	Asymptomatic	Rt. palm pain, Lt. finger pain, Lt. foot pain
Associated condition	PHP type 1A	PHP type 1A	PHP type 1A	PPHP
Genetic abnormalitiy	Heterozygous insertion of exon 5	Heterozygous deletion of exon 2	-	-
Brain lesion	O	O	-	-
Surgical removal	O	O	-	-
Histologic finding	Osteoma cutis	-	Osteoma cutis	Osteoma cutis

We have indicated with a “-” for sections where such information is not present.

## Data Availability

Data sharing is not applicable to this article because any datasets were made or analyzed during this study.
